# Rethinking language, cognition and assessment in psychosis: How bilingualism challenges psychiatry and how natural language processing can help

**DOI:** 10.1038/s41537-026-00742-1

**Published:** 2026-03-14

**Authors:** Sandra Anna Just, Vincent DeLuca, Jason Rothman, Brita Elvevåg

**Affiliations:** 1 Department of Clinical Medicine, UiT—The Arctic University of Norway, Tromsø, Norway; 2 Department of Psychiatry and Neurosciences, Campus Charité Mitte, Charité—Universitätsmedizin Berlin, Corporate Member of Freie Universität Berlin and Humboldt Universität zu Berlin, Berlin, Germany; 3 Department of Language and Culture, UiT—The Arctic University of Norway, Tromsø, Norway; 4 School of Social Sciences, University of Lancaster, Lancaster, UK; 5 Nebrija Research Center in Cognition, Madrid, Spain; 6 The Norwegian Center for Clinical Artificial Intelligence, University Hospital of North Norway, Tromsø, Norway

**Keywords:** Psychosis, Neuroscience

## Abstract

Language plays a central role in the assessment of individuals with psychosis, from taking a medical history to evaluating cognitive function. However, speaking multiple languages can significantly influence linguistic, cognitive and neural substrates. Therefore, it is essential to know whether an individual with psychosis is bilingual. Leaving modulating effects of bilingualism in psychosis unconsidered, runs high risk of confounding any clinical assessment and research. Although more than half of the world is bilingual, to date, this risk has not been addressed. This critical review challenges current basic diagnostic practices in psychiatry that conflate language and other cognitive domains. Drawing on neuropsychology, psycho-/neurolinguistics, and cognitive neuroscience, we (i) identify potential contact points between bilingualism and psychosis, (ii) present a decision tree framework for the clinical and research setting to systematically study those contact points, and (iii) provide the basis for developing and testing new treatments considering the lived realities of the majority of individuals with psychosis, namely bilingual individuals, and leveraging modern technology to do so. If the field of psychiatry embraces these conclusions, not only could bilingual individuals with psychosis experience more equity, but the larger field would benefit by reducing confounds inherent to ascribing to monolingual assumptions.

## Introduction

Psychiatry has a long tradition of investigating psychosis through speech^1^—recently with methods of natural language processing (NLP)^2–5^ to identify “linguistic markers” of symptoms and illness progression^6,7^. Even though bi-/multilingualism is the norm in many parts of the world^8^, and bilingualism can shape both linguistic behavior and cognitive performance^9–12^, to date, psychosis research has not systematically examined the bi-/multilingualism of affected individuals. This is astonishing given that essentially every assessment in psychiatry depends on language and that language/cognition is at the core of understanding psychosis^13–17^. Continuing to treat bilingual individuals with psychosis as monolingual or excluding them from research, without knowing how assessment and subsequent treatment may be biased by this action, is not only potentially harmful but discriminatory.

There is a very good reason to hypothesize an overlooked interaction between bilingualism and psychosis due to a considerable overlap in underlying mechanisms. Psychosis^18–21^ and bilingualism^10,22–24^ are complex, multidimensional phenomena, meaning the individual experience of each condition is shaped by a unique constellation of variables. The population of people living with psychotic disorders is highly diverse—differences occur by, but are not limited to, age, gender, remission status, and illness stage. Similarly, bilingualism is now understood as a continuum, determined by various factors, including how individuals engage with their languages. While bilingualism has been argued to alter brain structure and cognitive function^9,25–31^, research in recent years convincingly shows that it is the degree of language engagement that shapes linguistic and neurocognitive adaptations observed at the individual level^10,32–38^. To illustrate the issues at hand, Box 1 shows hypothetical vignettes of bilingual individuals experiencing psychosis.

A framework is introduced to guide research into whether and how bilingualism interacts with psychosis regarding outcomes in cognition and speech. Since, at present, no such framework exists, we do not know whether or how bilingualism affects cognitive performance and disordered speech in psychosis. Two lines of reasoning motivate this approach. First, evidence of underlying cognitive and brain mechanisms from bilingualism and psychosis points to a likely overlap between them, suggesting that the two may interact in ways that affect an individual’s neurodevelopment and their cognitive and linguistic abilities. Second, bilingualism is as ubiquitous as it is central to speech and cognition, which, in turn, are at the heart of diagnosing and monitoring patients. Without considering language background in cognitive assessments, the effects of language and other cognitive domains are conflated—e.g., when reduced performance in a verbal fluency task is attributed to executive dysfunction rather than a smaller vocabulary in the language of testing.

The proposed framework aims to improve the accuracy in the diagnosis of psychosis and improve treatment for bilinguals and monolinguals alike. Moreover, it will increase the validity of the results of intervention trials, pharmacological or otherwise, targeted at cognitive and speech impairments in psychosis. The framework also supports the broader move toward personalized medicine in psychiatry^39,40^ that should tailor assessments and treatments to a person’s language background. Thus, in this critical review, we argue how to (i) refine the interpretation of diagnostic assessments, from individual psychotherapy to multi-center pharmacological trials, and (ii) facilitate more personalized treatment approaches, while (iii) exploring the efficacy of modern technology to do so. While there are important distinctions between bilingualism (knowledge of two languages) and multilingualism (knowledge of more than two languages) for language representation and performance, linguistic processing, and potential adaptations to neurocognition^41^, for ease of exposition, we use the term bilingualism as a catch-all herein, as the main reasoning of our argumentation applies in all cases.

Box 1 Three hypothetical vignettes on bilingualism and psychosis **Vignette 1: Cognitive assessment beyond monolingual norms**

A 75-year-old bilingual woman with schizophrenia shows severe cognitive impairment on English cognitive tests but better performance in Spanish. Her psychiatrist is unsure whether to pursue dementia workup or if language factors explain the discrepancy. Research using our framework could develop test score accommodations based on bilingual language usage patterns. This would help clinicians distinguish between cognitive decline and language-related assessment confounds.

**Vignette 2: Leveraging bilingualism in treatment**

A young Syrian refugee experiences first-episode psychosis in Germany. While he communicates adequately in German, his doctor struggles to assess his emotional state during their sessions. However, with a bilingual psychotherapist, he can express complex emotions and thoughts in Arabic. Research using our framework could explore how different languages engage emotions differently during psychotic episodes, e.g., how language switching might regulate emotional states. This could inform new therapeutic approaches.

**Vignette 3: Compensatory effects of bilingualism on cognition**

A lifelong Mandarin-English bilingual man develops schizophrenia. His doctors note his remission pattern and cognitive performance differ markedly from typical cases. Our framework could help determine if bilingual experience is associated with better cognitive performance in psychosis. This might inform new cognitive training programs focused on language skills.

**Vignette 4: Research methodology and bilingual confounds**

A Canadian research study on psychosis found no overall group differences in cognition, but two sites showed significant outlying patterns. A post-doc discovered both outlier sites had twice the immigrant population of other sites. Since other factors were controlled, bilingualism may explain these differences. Many studies may miss important patterns by not considering bilingual language experience in individuals with psychotic disorders.

## Why is it relevant to study bilingualism in psychosis?

Beyond its core linguistic components (lexis, syntax, phonology, semantics, morphology), language processing depends on cognitive functions that are frequently implicated in psychosis, including cognitive control, discourse pragmatics, social cognition, and sensorimotor processes^14,42,43^. These cross-domain dependencies are well established in the monolingual and bilingual language processing literatures in general, but are perhaps most strongly reinforced in studies of impairment. For example, multilingual savants may show profound cognitive and communicative difficulties while retaining exceptional multilingual abilities^44^, whereas individuals with aphasia or developmental language disorder can exhibit severe linguistic deficits despite largely intact cognition^45–47^. For the present critical review, we therefore treat language both as (i) a distinct cognitive domain that may be shaped by bilingual experience and psychosis, and as (ii) the primary medium through which clinicians evaluate psychopathology.

### Bilingualism and psychosis overlap in relevant brain and cognitive functions

In order to identify potential contact points between psychosis and bilingualism, it is necessary to review the cognitive processes and associated brain functions that are affected by both the course of psychosis as well as the experience of engaging with multiple languages, and thus may interact over the lifespan of a bilingual individual with psychosis.

#### Neurocognition in psychosis: trajectories, affected domains and brain functions

The development of primary psychotic disorders such as schizophrenia is driven by neurodevelopmental and environmental factors^48–50^, with first signs occurring before the prodromal (initial) phase of the disorder. Early neurodevelopmental risk factors include brain differences between young children who are later diagnosed with schizophrenia and their neurotypical peers, affecting brain volume and functional connectivity, particularly in the prefrontal cortex and salience network^51–53^. These brain anomalies underlie later observed cognitive problems and increase the risk for psychosis^17,48,52^. The interplay between neurodevelopmental and environmental risk factors, such as adverse life events and substance use, places affected individuals on the psychosis continuum, with schizophrenia on ‘the severe end of a broader multidimensional psychosis spectrum’^48^.

Cognitive impairment is among the early signs of primary psychosis, occurring even in childhood^54,55^, progressing into a central symptom of the illness^14,16,56–58^, and negatively influencing prognosis and everyday function^59,60^. Moreover, changes in cognition likely mediate how pathophysiology and genetics contribute to the development of psychotic symptoms^14,61^, and affect multiple cognitive domains. Longitudinal studies confirm that cognitive impairment emerges early and persists. Data from a UK birth cohort study showed that only individuals who later developed a psychotic disorder showed increasingly lower scores in intelligence tests, not those with other mental disorders^55^. Similarly, a meta-analysis revealed a 0.4 standard deviation reduction in IQ scores in children who later developed schizophrenia^62^. Other studies found that the cognitive performance of children later diagnosed with schizophrenia remained consistently lower compared to healthy peers^55,63^. This well-documented pattern of poorer cognitive performance likely causes problems for affected children and adolescents during school years and when faced with more complex challenges in their everyday lives^63^.

The prodromal stage of schizophrenia typically emerges in adolescence or early adulthood. Notably, most individuals who display prodromal symptoms do not develop primary psychosis. This highlights that progression to primary psychosis is not inevitable but modulated by numerous factors^57,64,65^. Among those who progress to schizophrenia, cognitive performance during the prodromal stage is generally lower as compared to both healthy peers and prodromal individuals who do not develop schizophrenia^66^. Impairments affect all cognitive domains and have been associated with brain volume and connectivity differences, particularly in areas such as the prefrontal cortex and hippocampus, when compared to neurotypical peers^51,67–72^. Yet, no clear biomarker has been identified, underscoring the complex interplay between brain, cognition, and other risk factors^48,73,74^.

The transition from the prodromal stage to first-episode psychosis is marked by the greatest cognitive decline in the course of the illness, especially affecting memory, executive function, and processing speed, though all cognitive domains are affected, implying a generalized reduction in cognitive function^66,75,76^. Impairment of executive functions, such as planning, monitoring, problem-solving, and cognitive flexibility, appears to be particularly affected in schizophrenia, contributing to reductions in other cognitive and psychosocial functions^71,77,78^.

Individual trajectories of cognitive function in adults with schizophrenia vary—from further cognitive decline and stable impairment to improvement—and they appear to be modifiable^57,75,79–81^. While there is no unequivocal evidence supporting neurodegeneration in psychosis^82^, this does not rule out cognitive decline in later life. Some older individuals may experience a decline that exceeds healthy aging^57,83^. Contributing factors likely include high rates of metabolic syndrome, long-term antipsychotic use, and elevated cardiovascular risk^84–86^ as well as modifiable risk factors such as poor diet, physical inactivity^87^, and reduced cognitive stimulation (e.g., due to unemployment or disruptions in education)^57,84,85^. Importantly, the extent to which an individual is compromised by cognitive impairment in daily life is also shaped by premorbid cognitive abilities, social cognitive skills, motivation, and personal beliefs. Indeed, some individuals may be able to compensate for the loss of cognitive function^14^. Ultimately, the malleability of cognitive trajectories in schizophrenia underscores the potential value of interventions targeting modifiable lifestyle factors.

#### Neurocognition in bilinguals: which domains are affected?

The very act of using two (or more) languages requires neural and cognitive adaptations to meet an individual’s cognitive demands of managing multiple languages. Although bilingualism itself varies by degree of engagement with dual language exposure and usage, nonetheless there are overlapping neurocognitive outcomes with psychosis. The general mechanisms involved are likely the same irrespective of age or type of bilingualism (e.g., early childhood bilingualism versus adult sequential bilingualism commencing in adulthood), and dependent upon the actual engagement of the relevant cognitive processes necessary to manage two or more languages. Nevertheless, age differences can play an important role in bilingualism; for example, the cumulative experience of speaking multiple languages will increase as someone gets older, thus early bilinguals often have more experience. Moreover, earlier bilinguals are more likely on average to have a greater need for engaging language control, given the reality of their sociolinguistic circumstances (as opposed to an adult second language learner bilingual). Nevertheless, such differences are, in a sense, epiphenomenal to the reality of individual degrees of engagement at any given time operating over the same underlying mechanisms. Thus, differently from above, this section will not delve into observational differences by lifespan stage but rather focus more on the common mechanisms themselves.

By virtue of communicative necessity, it is argued that all languages a bilingual person knows are held at a certain level of resting activation so that they can be easily and rapidly selected as needed^28,88^. This joint activation requires the brain to actively select the target language and inhibit the unneeded language to avoid intrusions during communication, a set of processes termed language control^89^. Moreover, the cognitive processes and neural networks recruited for language control overlap with those used for domain-general cognitive functions, including attentional control and several aspects of memory^90,91^. The repeated and intensive engagement of language control necessitates specific neural adaptations to more effectively handle these demands, which have knock-on (typically positive) effects on the related cognitive processes.

A sizeable body of research supports the notion that bilingual experience contributes to adaptations in a variety of neurocognitive outcomes^92–94^. At the level of neurophysiology these adaptations typically manifest as both increases and reductions in gray matter in cortical and subcortical structures implicated in language- and domain-general control, including the inferior parietal lobule, anterior cingulate cortex, inferior frontal gyrus, caudate nucleus, thalamus, and hippocampus^95–99^; changes in white matter microstructure in tracts connecting these control regions^100,101^, and brain recruitment patterns in cognitive control tasks indicating increased efficiency^37,102–107^. Differences in trajectories across childhood and adolescence—specifically maintenance of gray- and white matter in language—and cognitive control related regions have been observed in relation to bilingual experience^30,108^, moreover degree of engagement with ones languages seems to calibrate the degree of this effect^109^. In older adults, brain regions and tracts implicated in language- and domain general control have been found to be reinforced against degrees of degradation associated with cognitive aging, such as the inferior parietal lobule, prefrontal cortex, hippocampus, inferior fronto-occipital fasciculus, superior longitudinal fasciculus, and corpus callosum^90,110–115^—see Gallo et al.^116^ for a review.

At the level of behavioral task performance, the existing data on the effects of bilingualism in younger adults show a positive effect on domain-general cognitive outcomes^92^. More specifically, bilingual experience has been linked to adaptations in performance on executive function tasks tapping into inhibitory control, task switching, working memory, and general intelligence^117–119^. Through development, bilingual experience has been associated with, typically, increases in task performance either compared to monolingual cohorts^120,121^ or in correlation with the degree of bilingual experience^122^. In older bilinguals, executive functions are often better maintained, inclusive of adaptations within the neural networks underlying them, providing a potential compensatory mechanism to the effects of cognitive aging, in the face of potentially increased (relative) neural degradation for the expected level of performance^29,123,124^.

It is important to note that, particularly in research comparing bilinguals to matched groups of functional monolinguals, these effects can appear inconsistently^125–127^. However, the main driver of the inconsistent effects seen in recent meta-analyses is variability across different studies, particularly in those using a dichotomous (e.g., bilingual versus monolingual) comparison^128^. Indeed, variability across studies is to be expected when considering the dynamicity and variability of bilingual experience. Individuals can differ on a number of axes, including (but not limited to) how long one has been bilingual, the degree of engagement with the languages, and the nature and degree of switching between languages. Given this, the contemporary approach rethinks the operationalization of bilingualism from absolutive to a continuum of its component experiences^12,22,128–130^. In tandem, an increasing number of theoretical proposals provide precise predictions about how specific aspects/degree of bilingual experience carry differing requirements on adaptations, and thus would correspond to distinct, individual neurocognitive outcomes^25,27,29–31^. The question has become not if bilingualism induces adaptations, but rather what are the conditions of bilingualism that correspond/calibrate to quantifiable changes^92^.

In support of this shift, a growing body of empirical work presents data indicating that distinct aspects of bilingual experience differentially modulate the nature and trajectory of neurocognitive adaptation. These experiential factors include (among others) the contexts of language exposure, intensity of engagement with both one’s languages, duration of (bilingual) experience, the nature and degree of switching between languages, and language proficiency^10,33–35,37,38,77,104,107,122,131–141^. Crucially, two trends have emerged within this literature. First, different experiences (e.g., duration or intensity of experience) correspond to distinct neurocognitive adaptations associated with an optimization towards handling the cognitive demands associated with that experience. Second, the degree of adaptation seems to be calibrated to the degree of that experience. While most of the evidence to date examining individual difference effects in the neurocognition of bilingualism comes from young adults, empirical work in older adults and in children suggests that similar predictive validity exists across the lifespan.

#### Overlaps between psychosis and bilingualism

This short summary of the literature on brain and cognition in primary psychosis and bilingualism points to a salient overlap between the two: executive control and associated brain regions are found to be impeded in individuals with psychosis (compared to healthy controls) and heightened in (some) bilinguals (compared to monolinguals). We therefore propose that *bilingualism should not merely be considered one of many factors introducing heterogeneity in psychosis, but a central one*. While we cannot claim to know the consequences of the interplay between bilingualism and psychosis yet, multiple possibilities can be envisioned. These range from no observable to compensatory effects. In bilinguals who actively use their spoken languages, one can expect to see adaptations, especially regarding executive control and its neural correlates^29,117,122,130^. Should such individuals go on to develop a primary psychotic disorder, it is plausible that the cognitive adaptations associated with bilingualism have interacted with the neurodevelopmental trajectory of the illness. As a result, active bilinguals with psychosis may demonstrate different cognitive outcomes than passive bilinguals or monolinguals with the same diagnosis. It should be noted that cognitive compensation may be beneficial in some contexts, but can also risk misdiagnosis or inappropriate treatment—as for bilinguals in other illnesses affecting speech^142,143^.

### Bilingualism shapes speech and cognition, which are central to symptom assessment in psychosis

#### The role of language and bilingualism in psychiatric assessment

In psychiatry, every formal mental status assessment depends on spoken or written language – no diagnosis is solely based on neuroimaging or blood analysis. The clinical interview exemplifies two things, namely (i) how language is used to directly gather information about a patient’s health status while (ii) simultaneously containing information about symptoms expressed through speech^144–146^. Regarding the first issue (i), clinicians use speech to inquire about various aspects of patients’ mental health (e.g., mood, drive, thoughts, concentration), while patients use speech to describe their symptoms (e.g., depressed mood, low drive, ruminating thoughts, concentration difficulties). Regarding the second issue (ii), the pattern and content of the communication of an individual with psychosis provides large amounts of information that can be traced back to their overall mental state. For instance, a patient may be taciturn, use very few words, and take a long time to respond, pointing to numerous potential differential diagnoses, including depression, schizophrenia, dementia, or, alternatively, a lack of cooperation. Thus, in (ii), language serves as an indicator of underlying cognitive and mental processes, a measurable, clinical sign^147^. Cognitive impairment not only affects the patient’s ability to report symptoms (e.g., due to difficulties in remembering them correctly) but may itself manifest as a symptom in unusual language patterns (e.g., formal thought disorder, aphasia)^148,149^. This may explain the focus of clinical rating scales for psychosis on linguistic symptoms^150^ and is reflected in the very term formal *thought* disorder: the assumption that language is a direct window into the mind.

What does this mean for bilingual individuals who undergo psychiatric and cognitive evaluations? Does it matter in which language the clinician asks the questions and which of their languages a patient recounts their experiences to their clinician? To address this, we first outline the bilingual language mechanisms most relevant to psychiatric assessment, before turning to their implications for assessing bilingual individuals with mental illness (see Table 1 for examples of potential biases).

**Table 1:** Examples of biases in the assessment of bilingual individuals and their impact.

	Sources of bias in bilinguals	Examples of potential impact of bias
**Vocabulary**	Smaller, more specific vocabulary in each language; larger gap in receptive vs. productive vocabulary.	Decreased fluency may be misinterpreted as cognitive impairment or psychopathological symptoms such as poverty of speech.
**Foreign-language effect**	Less emotional responses in non-dominant language, and more utilitarian in decision-making.	Assessments in only one of the languages may fail to chart the full extent of psychopathology. Patients may arrive at different decisions regarding their treatment depending on the language context.
**Norms**	Culturally inappropriate test materials; interpretation of measurements from bilingual individuals based on monolingual norms.	Incorrect clinical decisions if language behavior is falsely attributed to pathology or masks underlying pathology.
**Assessment language**	Assessment in non-dominant language of either or both patient and clinician; using interpreters.	Difficulties in articulating or understanding nuanced emotional and mental states in non-dominant language can negatively influence therapeutic alliance, symptom detection.

Speaking more than one language affects how people understand, produce, and process language across all linguistic domains. These effects vary widely depending on the type of bilingualism and individual factors such as age of acquisition, proficiency, exposure, and usage. However, a few consistent observations are relevant for the language assessments typically used in psychiatry. While bilinguals do not have smaller vocabularies in general (considering all lexicons they command), their lexical inventories in each language are overall smaller and—for languages they are less dominant in—more specific to the functions in which they are used (e.g., rich lexical knowledge of vocabulary pertaining to their occupation)^151,152^. Moreover, the gap between receptive and productive vocabulary tends to be larger in bilinguals than monolinguals^153^—meaning they understand more words than they actively use. It is likely that a bilingual speaking their non-dominant language, relative to dominant native speakers of the same language, does not have the same breadth of lexical knowledge across all domains. And so, in either case, one might find bilinguals of all types to perform differently from the norms of dominant monolinguals on semantic naming tasks, reflecting more their experience with the language than anything related to differences in semantic networks.

Work on the so-called “foreign language effect” suggests that bilinguals do not make the same types of decisions when faced with similar issues in each of their languages, especially, or perhaps only true for non-sequential adult language learners, where proficiency can also modulate this effect. Research has shown that bilinguals are more likely to be increasingly utilitarian in their decision making and moral judgments and less emotive in their communication as it relates to their non-dominant language^154–157^, although the context in which such decisions or communication take place matter^158^. It should also be noted that emotions and how people express them can differ across different languages and cultures, in monolingual and bilingual individuals^159^. These findings on bilingualism have important implications for psychiatric assessments. From obtaining a patient’s clinical history through open conversation and collecting diagnostic information through interviews and questionnaires to measuring cognitive abilities—language is the medium of assessment. Even people who are highly fluent in their non-dominant language(s) may find it difficult to express the intricacies of their inner world in that language^160^ or at least express themselves differently compared to when using their dominant language^161,162^. When, in addition, they have a serious mental illness such as psychosis that compromises their ability to think and speak, it seems likely that results from clinical assessment may differ between their languages. Moreover, even when testing occurs in a patient’s dominant language, the use of monolingual norms introduces biases. Research on developmental language disorder indicates that monolingual norms can lead to misdiagnosis^143^, while in dementia, bilingualism can mask the underlying pathology^142^. These examples urge caution regarding the use of monolingual norms for assessing language-related conditions in bilingual individuals.

Cognitive tests developed for a specific population (often White monolingual) are not representative of other backgrounds. One consequence is that test materials may be understood differently by individuals outside that context, regardless of proficiency^163,164^. Additionally, interpretation of test scores may be impeded when a monolingual clinician conducts cognitive assessments in a language they do not speak proficiently or command equally well as their dominant language. The use of interpreters introduces its own challenges, as the prevalence of reported or detected symptoms can differ significantly between psychiatric assessments supported by interpreters and those conducted in a patient’s dominant language^165^. Despite guidelines for working with interpreters, translation can still introduce bias^166^.

Given the central role of language in psychiatric assessments, especially in psychosis, evaluating bilingual patients is inadequately addressed by translating instruments or using interpreters. As the language of assessment influences results, psychiatric and cognitive evaluations should always consider not only an individual’s status as a bilingual per se, but crucially the insights and implications of research on bilingual language and neurocognition.

#### Assessment of bilingual individuals with psychosis

A recent scoping review^150^ on psychosis in bilinguals found that most research focused on the assessment of symptoms across languages. Some patients reported more symptoms in their dominant language, others fewer^150,167–169^. A meta-analysis on bilingual psychosis reported a 3–30% higher probability of detecting symptoms in the dominant language, while noting a low quality in available studies^170^. Another review reported that patients experienced more symptoms and were more emotionally involved in their dominant language, but that some studies documented opposite results^167^. Whether symptoms were truly reduced in the non-dominant language, underreported, or not detected remains unclear. Clinical reports from psychoanalysis^171^ propose that the native language is more emotionally charged, while the second language allows emotional distance. This aligns with findings that patients are more open in interviews in their native language^168,172^—although it is easy to speculate about other reasons, such as feeling more comfortable with a clinician of the same language background. If confirmed, bilingual psychotherapy for patients with psychosis could utilize language switching to modulate emotional engagement in the individual course of recovery^173,174^.

Psychosis researchers are aware that language background may affect cognitive performance, but the field has not examined this influence systematically. Instead, studies either exclude bilingual or non-native speakers^175–177^ or do not collect detailed information on language history. A meta-analysis on cognition in first-episode schizophrenia^75^ reported that only 2% of 47 studies controlled for country of birth—an insufficient proxy for bilingualism but an indicator that the sample may not have been monolingual. Even when participants were born outside the study country, studies have reported on language background, proficiency, or assessment language^178,179^. As a result, a strong case can be made that current research merely touches the surface of bilingualism in psychosis.

## A framework to study bilingualism and psychosis

**Fig. 1:**
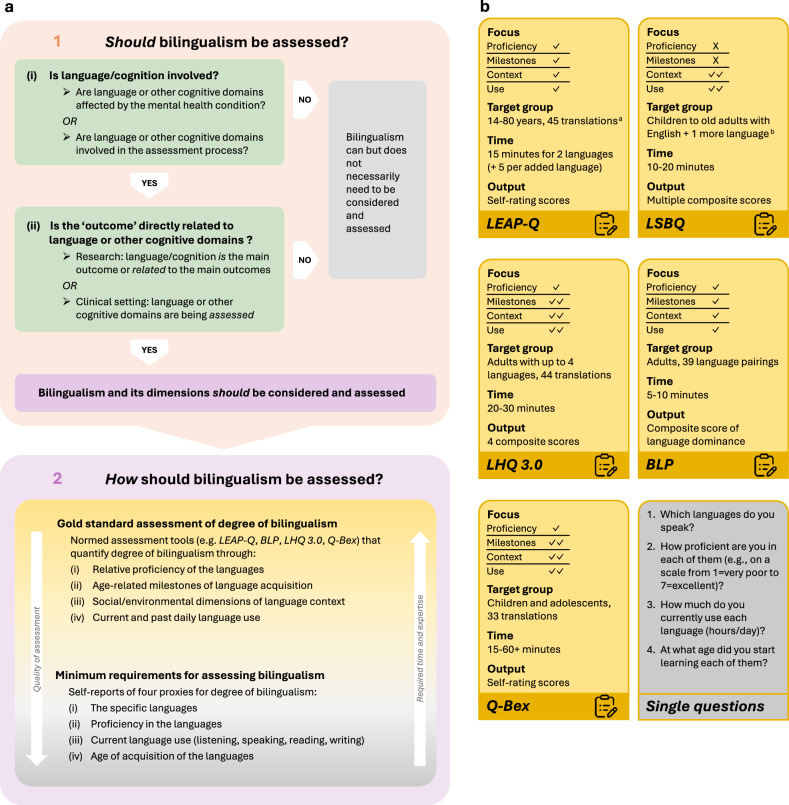
A decision tree framework for deciding whether and how bilingualism should be assessed in mental health research and practice. **a** Clinicians and researchers are guided by two questions: 1. Should bilingualism be assessed?, and if yes, 2. How should bilingualism be assessed? To answer Question 1, one should ask whether language or other cognitive domains are relevantly involved in one’s research or practice, either because language/cognition is affected by the mental health condition (e.g., psychosis) or because language/cognition is involved in the assessments one is planning to conduct (which is usually the case in psychiatric assessments). If language and/or other cognitive domains are deemed to be relevant, one should ask next whether the “outcome of interest” in one’s practice or research is directly related to language/cognition. In research, language/cognition can be the main outcome (e.g., when examining speech changes in psychosis) or it can be significantly related to the main outcome. In the clinical setting, language and/or other cognitive domains are considered the “outcome” when clinicians assess their patients’ linguistic or cognitive abilities (a wider interpretation is discussed in the text). If language and/or other cognitive domains are deemed to be the “outcome” or related to it, bilingualism should be assessed. If they are not deemed relevant, bilingualism still could but does not need to be recorded. Question 2 asks how bilingualism should be assessed and guides researchers and clinicians through recommendations on a gradient from the gold standard of assessing the degree of bilingualism to minimum requirements. **b** Standard assessment tools are compared with regard to the degree that they measure (i) “proficiency”—the relative proficiency of the languages, (ii) “milestones”—age-related milestones of language acquisition, (iii) “context”—social and environmental dimensions of language context, (iv) “use”—current and past daily language use. “X” indicates that this aspect is not directly assessed. “✓” indicates that the aspect is directly assessed, and “✓✓” indicates significant detail. “Target group” describes which ages the tool is available for, as well as ^**a**^ translations (i.e., the number of languages the tool has been translated to) or ^**b**^ languages/language pairings (i.e., the number of languages that are covered by the tool, regardless of translation). “Time” indicates the estimated duration needed to administer the assessment tool. “Output” lists which scores the assessment produces. “Single questions” are suggestions on how to meet the minimal requirement for assessing language background. LEAP-Q Language Experience and Proficiency Questionnaire^182,183^, LSBQ Language and Social Background Questionnaire^184^, BLP Bilingual Language Profile^185^, LHQ 3.0 Language History Questionnaire 3.0^186^, Q-Bex Questionnaire of Bilingual Experience^187^.

Our review of the literature on neurodevelopment and cognition in psychosis and bilingualism emphasizes that (i) bilingualism and psychosis may interact in ways that influence illness expression and key clinical outcomes in psychosis, and that (ii) assessments based on (presumed) monolingual populations have limited applicability to bilingual populations and individuals. Whether conducting cognitive assessments in bilingual patients with psychosis in clinical settings or for research purposes, bilingualism must be systematically considered and examined. In the following, we introduce a framework for this purpose in both clinical and scientific contexts. The framework is developed as a decision tree (see Fig. 1) to support clinicians and researchers in answering two key questions:

First, s*hould* bilingualism be assessed?

Second, if bilingualism is to be considered, *how* should it be assessed?

### Should bilingualism be assessed?

When deciding whether bilingualism should be assessed in a clinical or research setting, we propose considering two questions:

(i) Is language/cognition involved—either because it is affected by the mental health condition or because it is involved in the assessment process?
  In the clinical setting, specifically for assessments around psychotic disorders, the answer to the first question will usually be “yes”. Changes in language and other cognitive domains are at the core of psychosis; they are involved in any psychiatric assessment and will differ between monolingual and bilingual individuals. Indeed, it is difficult to imagine any mental disorder that does not involve language/cognition, given its centrality to the human experience. However, our premise is that the relationship between bilingualism and psychosis is not just a superficial overlap, but that there is a deep interaction that may alter the underlying mechanisms of both.
(ii) Is the “outcome of interest” directly related to language or other cognitive domains?

In the scientific context, this question can be understood in both a narrow and a broad sense. A narrow interpretation asks whether language/cognition is the primary focus of research. This applies to studies that assess cognitive functions such as memory or executive functioning, or that analyze speech and language. In such cases, bilingualism should be assessed, as it directly affects the outcome. Studies that focus on outcomes not directly related to language/cognition, such as socioeconomic status, trauma history, or interpersonal relationships, would not have to consider bilingualism. Yet, when applying a broader understanding, bilingualism may still be relevant—either because it indirectly influences outcomes via its impact on language/cognition, or because these outcomes shape how bilingualism itself manifests. For example, bilingualism may be relevant for studies examining socioeconomic status, as higher linguistic and cognitive abilities can support success in education and employment. Conversely, socioeconomic status is also associated with cognitive and linguistic abilities, potentially interacting with bilingualism in complex ways^180,181^. Therefore, if both questions are answered yes in a narrow sense, bilingualism should be assessed. If the second question is answered positively only in the broader sense, bilingualism can be considered, but this is not strictly required.

In the clinical context, answering the second question is more complex, as the concept of “outcome” is less defined. When conducting standardized assessments of clinical or linguistic abilities, clinicians should be aware whether their patient is bilingual and monolingual norms are applicable. Otherwise, the results of the assessment will not be reliable, and there is a risk that clinicians under- or overestimate their patients’ linguistic and cognitive abilities. The question is whether this also applies to non-standardized open conversations with patients that inform clinicians, nurses, and therapists about their patients’ current mental state. Here, the “outcomes of interest” are less defined and quantifiable, but equally important. While clinicians cannot compare a conversation’s impression to tables of normative data, they will form an interpretation based on their own sociocultural and linguistic background. Empathy and personalized care in this situation require understanding all factors that shape the patient’s experience—bilingualism included. Therefore, clinicians should ask about patients’ language histories and not stop at proficiency alone. Although not mandatory, it will be beneficial for their patients. However, when conducting assessments, clinicians should consider whether the normative data are appropriate for their bilingual patients.

### How should bilingualism be assessed?

If researchers and clinicians answer affirmatively both questions regarding the relevance of bilingualism for their participants or patients, they should aim to consider and assess bilingualism in their work. However, given that most clinical researchers or practitioners will not have formal training in language sciences, this poses a practical challenge. To address this, our decision tree framework provides recommendations on which core variables of bilingualism must be assessed to adequately control for confounding between language and other cognitive domains, and which additional aspects of language background, while not essential, may enhance the depth and quality of psychiatric research.

Given that degree of bilingual engagement and language usage patterns are currently understood to predict and calibrate to each individual’s resulting neurocognitive adaptations, the ideal recommendation is to undertake an exhaustive assessment of language background. This should include a measure that can gauge relative language proficiency, chart key age-related milestones to the acquisition of the languages, offer insights into the social/environmental dimensions of the languages in context, and, crucially, offer a breakdown of how the two (or more) languages are and have been used on a daily basis in real and apparent time. Figure 1b describes several normed assessment tools that are suitable. Among the most commonly applied and user-friendly tests are: (i) Language Experience and Proficiency Questionnaire (LEAP-Q)^182,183^; (ii) Language and Social Background Questionnaire (LSBQ)^184^; (iii) Bilingual Language Profile (BLP)^185^; (iv) Language History Questionnaire 3.0 (LHQ 3.0)^186^, and Questionnaire of Bilingual Experience (Q-Bex)^187^. For an in-depth discussion of these main assessments (and others) used in the bilingualism literature, we refer the reader to Rothman et al.^188^ where they are discussed in rich practical detail, as well as Dass et al.^189^ where their overlap and distinctions in coverage are assessed. For the present purposes, what is important is that each provides an in-depth assessment of how the languages came to be acquired and what their distributions are to quantify an individual’s degree of bilingualism. These assessments vary in terms of what they cover and focus on. For example, some are more concerned with computing relative dominance between languages (e.g., BLP). Few have been explicitly designed to properly consider questions related to language attitudes and ideologies (Q-Bex) beyond usage patterns in context. Some are limited to capturing true bilingualism (where two languages are involved, e.g., LSBQ), whereas others have been designed to quantify across multiple languages (up to 4, LHQ 3.0). And finally, some have been specifically designed for capturing language exposure and use in children and adolescents (Q-Bex), where different variables are likely at play. In all cases, the assessment tools compute either a single or various composite scores, offering continuous, relative quantification of the degree of bilingualism.

Naturally, the above assessments require an investment in time (30−60+ min). However, if bilingualism itself as an outcome is of interest, or there is a curiosity in how language history may inspire new research questions, or how bilingualism might manifest important considerations for individuals, then these additional assessments are likely to be enormously useful. If bilingualism is not the focus of research, such extensive assessments are difficult to justify, as participants with mental illness should only be subjected to assessments that are proportionate in burden. For these cases, and the clinical setting, it can be feasible to assess proxies of the degree of bilingualism, using quicker and, by default, simpler metrics. These can include: (1) recording the specific languages in an individual repertoire and (2) asking for a self-reported (relative) proficiency in the languages, (3) amount of daily/regular engagement with the languages across the dimensions of listening, speaking, reading and writing, and (4) age of acquisition of the languages (that is their age at first exposure from which point on they learned the language). Both proficiency and engagement/use can be asked with a defined Likert scale, and asking across the four domains of language (listening, speaking, reading, writing) will also reveal if an individual is literate in the language. Age of acquisition can be used as-is or subtracted from the individual’s age to gauge a general duration of exposure to the languages, as well as determine what type of bilingual one is, i.e., simultaneous child bilingual or a late sequential language learner.

## Future trends

Numerous research questions can be derived from our framework, as detailed in Table 2. One promising direction to scale bilingualism research is to leverage modern methods from Artificial Intelligence (AI).

**Table 2:** Potential research questions on psychosis and bilingualism.

Topic	Examples for research questions
**Group comparisons**	How do monolingual and bilingual individuals with psychosis (or other mental health conditions that may interact with bilingualism and affect language and cognition) differ in cognitive and speech outcomes? Do group differences vary by cognitive domain?
**Illness expression**	Do bilingual individuals with psychosis experience and report symptoms differently across languages? Does this difference reflect genuine variation in symptoms or in the language-mediated reporting biases?
**Language history**	How are cognitive and linguistic outcomes in psychosis shaped by different bilingual experiences, such as the number of years of active engagement with the languages, the context of language use, age of acquisition, or language pairings?
**Therapeutic leverage of bilingualism**	Can therapists and patients modulate emotional intensity by switching languages? Can strategic language switching be utilized by patients and/or therapists in support of recovery in psychosis?
**Sociocultural influences**	How does bilingual identity intersect with illness trajectories and recovery? How can an individual’s language and cultural identity be considered in their treatment?

The lack of research on bilingual psychosis is at least partly due to the inadequacy of traditional methods, which rely on bilingual clinicians or interpreters—an approach that is costly, time-consuming, and simply not feasible on a large scale. With the vast number of global language combinations, it is nearly impossible to recruit enough multilingual professionals for large-scale assessment and research. As a result, only a limited set of languages is typically represented in research, while smaller and indigenous languages remain largely overlooked in psychiatric research.

AI methods, particularly automatic speech recognition and NLP, offer ways to automatically recognize and examine speech and thus present an efficient alternative to traditional assessments. They could enable cross-language comparison of speech features and automated identification of clinically relevant linguistic markers across both languages of a bilingual speaker. While the use of NLP methods has become a common approach in psychosis research, it appears to be relatively novel to the field of bilingualism, except for a few studies^190^. These technologies make it possible to include underrepresented languages and to study rare but increasingly relevant language pairings in a globalized world. Without such speech technologies, research at this scale would remain prohibitively expensive and logistically unfeasible.

However, the potential of AI-based tools and methods cannot only be leveraged for automatically analyzing language output (e.g., syntax, semantic associations, vocabulary) in different languages. Recent advances in generative AI and large language models (LLMs) present new opportunities for translating assessments as well for creating new, more personalized assessment approaches^191^. Modern technologies can also be used for creating more efficient ways of administering assessments. For example, LLM-powered chatbots could conduct structured interviews in multiple languages as well as administer and score tasks remotely.

By enabling research across a far broader linguistic spectrum, AI-based methods could potentially contribute to greater equity in mental health research, ensuring that the experiences of bilingual individuals, regardless of their language background, are appropriately studied and understood. However, it should be noted that AI only fosters equity if models are evaluated cross-linguistically and for multilingual scenarios; results from NLP analysis tend to differ between languages^192^ and can be biased, dependent on the size of a language^193^ as well as language background^194^. Still, enabling bilingualism research in clinical populations at all is a crucial and long-overdue step forward.

## Conclusion

The exact influence of bilingualism on the clinical presentation of psychosis remains an open empirical question that needs to be systematically examined. Given the identified contact points in the brain and cognitive mechanisms between bilingualism and psychosis and the centrality of language in psychiatric assessments, failing to consider bilingual language experience risks undermining the validity of both clinical assessment and scientific research on psychosis. The framework proposed here offers a path forward that recognizes bilingualism not as a confound but as a key variable in understanding individual differences in psychosis. This framework is not only necessary for accurate assessment of individuals with psychosis, but it is also necessary to ensure equity in care.

## Data Availability

No datasets were generated or analyzed for this article.
